# Risk analysis of the association between lactate-to-hematocrit ratio and poor prognosis in critical patients with cirrhosis

**DOI:** 10.1097/MD.0000000000049155

**Published:** 2026-06-05

**Authors:** Ya Chen, Yihuai He, Yunfen Chen, Longmin Qiu, Yawen Luo

**Affiliations:** a Department of Infectious Diseases, The Affiliated Hospital of Zunyi Medical University, Zunyi, Guizhou, China.

**Keywords:** cirrhosis, intensive care unit, lactate-to-hematocrit ratio, mortality, prognosis, risk stratification

## Abstract

Liver cirrhosis contributes substantially to global health and causes high mortality in critically ill patients. While the lactate-to-hematocrit ratio (LHR) has established prognostic value in diverse critical illnesses, its utility in critically ill cirrhotic patients remains underexplored. This study evaluated the role of LHR in predicting short- and long-term outcomes in this distinct clinical cohort. This retrospective study analyzed cirrhotic patients using the MIMIC-IV database. LHR was derived from initial arterial blood lactate (LAC) and hematocrit (HCT) measurements. Participants were stratified by LHR quartiles, with 30-day all-cause mortality as the primary endpoint and 180-/365-day mortality as secondary endpoints. The associations between LHR and mortality were assessed using Kaplan–Meier survival analysis and multivariable Cox regression models. Further determine whether there is a linear relationship using restricted cubic spline (RCS) curves. Finally, subgroup analyses were conducted to assess the consistency of the LHR-prognosis relationship across various patient subsets. In a cohort of 2998 critically ill cirrhotic patients, elevated LHR was strongly associated with increased mortality at 30, 180 and 365 days. Compared to the lowest quartile, patients in the highest LHR quartile had a 144.2% higher risk of 30-day mortality (hazard ratio [HR] = 2.442, 95% confidence interval [CI]: 1.951–3.057), a 139.6% higher risk at 180 days (HR = 2.396, 95% CI: 1.95–2.944) and a 139.1% higher risk at 365 days (HR = 2.391, 95% CI: 1.955–2.925). Kaplan–Meier survival analysis revealed significantly worse outcomes for patients with higher LHR at all time points (log-rank *P* < .001). Additionally, restricted cubic splines analysis showed a significant nonlinear relationship between LHR and both short- and long-term mortality risk. Subgroup analyses revealed that the association between LHR and mortality was heterogeneous across specific patient characteristics, with significant interactions observed particularly in those with spontaneous bacterial peritonitis and those using glucocorticoids. LHR is an independent predictor of short- and long-term all-cause mortality in critically ill cirrhotic patients. Given its simplicity and availability from routinely measured parameters, integrating LHR into early clinical assessment may facilitate timely risk stratification and support clinical decision-making in the intensive care unit.

## 1. Introduction

Cirrhosis is a chronic and progressive hepatic disorder marked by fibrosis and a progressive decline in liver function, stemming from persistent damage caused by factors including chronic hepatitis, excessive alcohol consumption and metabolic fatty liver disease.^[1–3]^ In its critical phases, the condition becomes life-threatening, with limited treatment alternatives and a profound impact on both survival and quality of life.^[4]^ Cirrhosis is accountable for nearly 2 million deaths annually worldwide, making it the 11th leading cause of mortality globally.^[5,6]^ As the illness advances, serious complications may arise, such as hepatic failure, encephalopathy, spontaneous bacterial peritonitis (SBP), gastrointestinal bleeding (GIB), hepatorenal syndrome and multi-organ dysfunction, often requiring intensive care unit (ICU) admission for advanced monitoring and life-sustaining interventions.^[7]^ These complications significantly elevate fatality rates and contribute substantially to the global health burden, especially in low- and middle-income countries.^[5]^

Cirrhotic patients admitted to the ICU often experience an unstable clinical course, characterized by multi-organ system dysfunction. Although conventional prognostic models, such as the Child-Pugh, MELD and SOFA scores, provide a basic risk assessment, their practical application in critical care settings remains constrained. These tools generally rely on a combination of several clinical variables, rendering them operationally cumbersome and less suitable for rapid evaluation in unstable patients. Limitations related to predictive accuracy, inter-observer variability and generalizability further restrict their ability to reliably forecast short- and long-term prognosis in this population.^[8]^ Hence, there is a pressing need to discover a clinically accessible and highly discriminative biomarker that can facilitate early and accurate mortality risk stratification, ultimately aiding in better clinical decision-making and improving outcomes in this high-risk population.

The lactate-to-hematocrit ratio (LHR) has gained attention as a potential prognostic biomarker in recent clinical studies.^[9,10]^ While initially validated as a predictor of mortality in patients with severe thoracoabdominal trauma^[9]^ and sepsis,^[10]^ the role of LHR in cirrhotic populations is not well characterized. To date, only one study has evaluated LHR for predicting 14-, 28- and 90-day outcomes and it was conducted specifically in cirrhotic patients with sepsis.^[11]^ Consequently, the prognostic value of LHR in cirrhotic patients without sepsis remains unclear.

Lactate (LAC), a key marker of tissue perfusion and metabolic status,^[12–14]^ is strongly associated with prognosis in critically ill patients.^[15–19]^ In the context of cirrhosis, impaired hepatic clearance often leads to lactate accumulation and can exacerbate metabolic acidosis.^[20,21]^ Clinical evidence confirms that initial lactate levels are significantly lower in cirrhotic ICU patients who survive 28 days (1.2–3.4 mmol/L) than in non-survivors (2.0–9.7 mmol/L), with survivors also demonstrating superior lactate clearance.^[22]^ A blood lactate exceeding 2 mmol/L within 24 hours of admission is a recognized risk factor for ICU mortality.^[23]^ Hematocrit (HCT) is a standard clinical indicator of oxygen-carrying capacity and anemia.^[24]^ A low HCT has been associated with unfavorable prognoses in several diseases.^[25,26]^ The liver plays a vital role in hematopoiesis, and its dysfunction in cirrhosis commonly results in anemia and erythropoietic impairment through multifactorial mechanisms.^[25]^ Studies indicate that the severity of anemia helps inform risk stratification and serves as an independent prognostic factor in HBV-related decompensated cirrhosis.^[27,28]^

Despite their established prognostic value, both LAC and HCT exhibit limitations when used individually for risk assessment in critically ill cirrhotic patients. LAC levels are nonspecific and can be influenced by various factors, including tissue perfusion and hepatic metabolic function. HCT is a dynamic variable, susceptible to alteration by fluid resuscitation, nutritional support and inflammatory states. The combination of these 2 routinely available parameters into the LHR represents a pragmatic approach to developing a composite biomarker. The LHR may thus provide a more integrated view of the patient’s metabolic, synthetic and perfusional status, potentially enabling a more accurate assessment of the complex pathophysiology in this population.

Based on this rationale, we performed a retrospective analysis to assess the prognostic value of LHR in predicting short- and long-term all-cause mortality in critically ill cirrhotic patients.

## 2. Materials and methods

### 2.1. Study population

**Figure 1.**
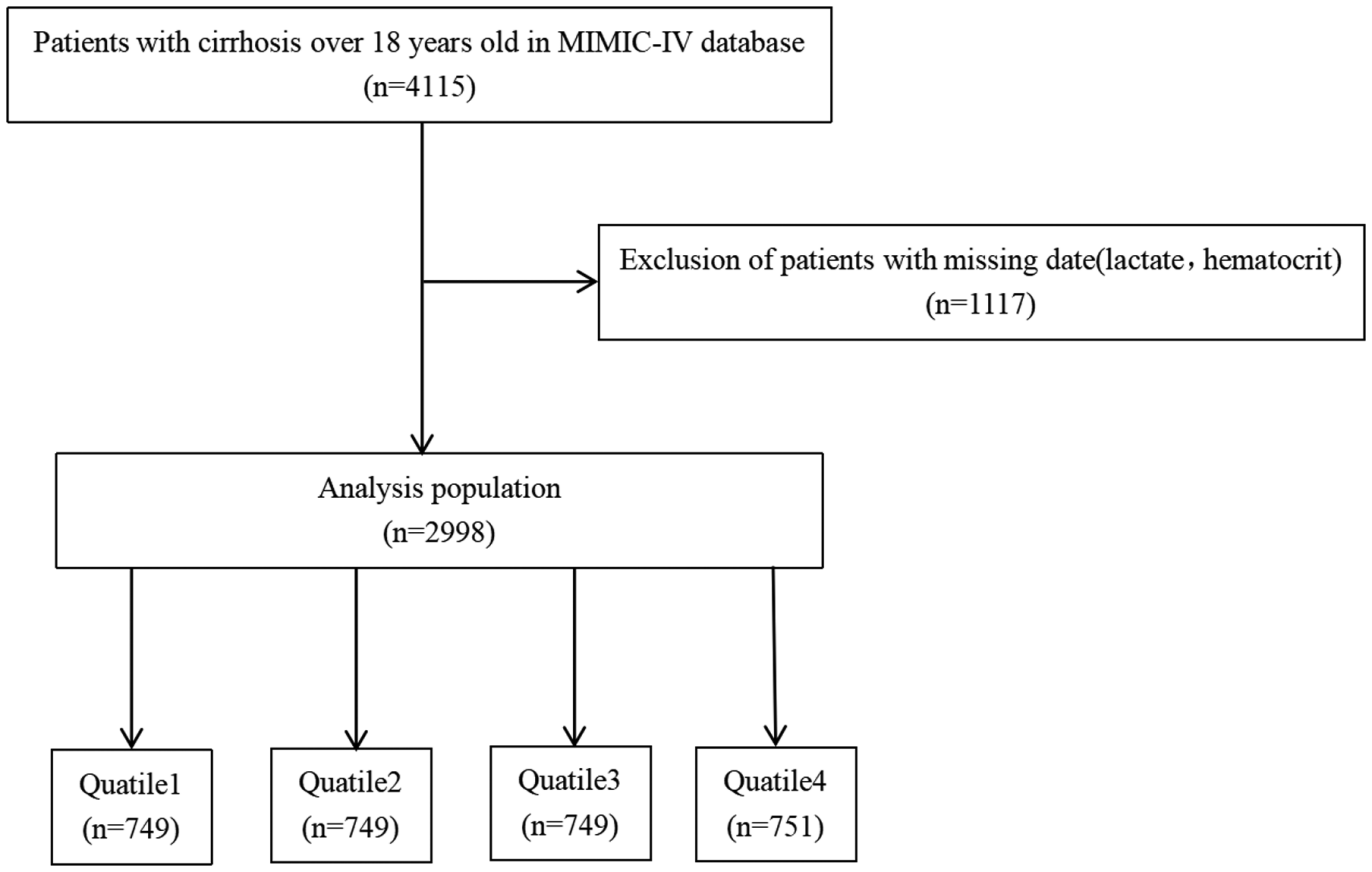
Flow of included patients through the trial.

This retrospective study utilized data from the MIMIC-IV (version 3.1) database, a publicly available repository developed and maintained by the MIT Computational Physiology Laboratory. This database contains detailed, high-quality medical records of patients admitted to the intensive care units of the Beth Israel Deaconess Medical Center.^[29]^ One investigator (Ya Chen, Record No:73859549) completed the required training and was granted access to extract the relevant data. Adult patients (age ≥ 18 years) with a diagnosis of cirrhosis, defined using relevant International Classification of Diseases (ICD-9: 5715, 5712, 5716; ICD-10: k7469, k7460, k7031, k7030, k743) codes, were initially considered. We excluded patients with multiple ICU admissions for cirrhosis (retaining only data from their first admission) and those with missing LAC or HCT data on the first day of admission. The final cohort comprised 2998 patients, who were subsequently categorized into 4 groups based on LHR quartiles (Fig. 1).

### 2.2. Data collection

Using DecisionLinnc 1.0 software,^[30]^ comprehensive admission data were extracted via structure query language. The potential variables were categorized into 6 groups: demographic characteristics, such as age, gender, weight and race; vital signs, covering heart rate, mean blood pressure (MBP), respiratory rate (RR), percutaneous arterial oxygen saturation (SpO_2_) and temperature; disease severity scores, including sequential organ failure assessment (SOFA), simplified acute physiology score II (SAPS II), oxford acute severity of illness score (OASIS), glasgow coma scale (GCS) and charlson comorbidity index (CCI); laboratory parameters, namely LAC, HCT, hemoglobin, platelet count, white blood cell (WBC), red blood cell (RBC), glucose, sodium, INR, prothrombin time (PT), ALT, AST, TBIL, creatinine and BUN; comorbidities, which consisted of renal failure, respiratory failure, hepatocellular carcinoma (HCC), hepatopulmonary syndrome, SBP, hepatorenal syndrome, GIB and heart failure (HF); clinical interventions, specifically antibiotics and glucocorticoids (GC). Patient follow-up started at admission and concluded at the date of death. The LHR was computed according to the formula: LHR = LAC (mmol/L)/ HCT (%) * 100%.^[9–11]^ All laboratory values and severity scores were collected within the first 24 hours of ICU admission.

To minimize potential bias, variables with more than 20% missing data were excluded, while those with fewer missing values were handled using multiple imputation.

### 2.3. Clinical outcomes

This study defined the primary outcome as all-cause mortality within 30 days after the first ICU admission. The secondary outcomes were all-cause mortality assessed at 180 and 365 days post-admission.

### 2.4. Statistical analysis

Continuous data are presented as mean ± standard deviation or median (interquartile range), depending on their distribution, while categorical variables are reported as frequencies and percentages. The normality of continuous variables was assessed using the Kolmogorov–Smirnov test. Group comparisons for continuous variables were made using the Student *t* test or 1-way ANOVA for normally distributed data and the Mann–Whitney *U* test or Kruskal–Wallis test for non-normally distributed data. Survival curves were generated using the Kaplan–Meier method according to LHR levels and compared using the log-rank test. To identify factors associated with all-cause mortality, Cox proportional hazards regression models were used. Hazard ratios (HRs) and 95% confidence intervals (CIs) for the association between LHR and outcomes were calculated in both unadjusted and adjusted models. Covariates for multivariable models were selected based on a univariate screening threshold of *P* < .05, as well as clinical relevance. Three models were constructed: Model 1 (unadjusted); Model 2 (adjusted for demographic factors: age, gender, weight and race); and Model 3 (further adjusted for age, gender, race, MBP, SOFA score, OASIS score, creatinine, glucose, INR, sodium, WBC, TBIL, hepatorenal syndrome, SBP and antibiotic use). The LHR was included as both a continuous and categorical variable, with the first quartile used as the reference. Additionally, we examined the potential nonlinear relationship between baseline LHR and ICU all-cause mortality using restricted cubic spline (RCS) regression with 3 knots. Trend analysis across LHR quartiles was performed. Subgroup analyses were conducted by gender, race, HCC, SBP and GC use to evaluate the consistency of LHR’s prognostic effect. Interaction effects were tested via likelihood ratio tests. A 2-sided *P* < .05 was considered statistically significant. All analyses were performed using DecisionLinnc 1.0,^[30]^ an integrated software platform that supports multiple programming languages and provides visualization tools for data processing, statistical modeling and machine learning.

## 3. Results

A total of 4115 cirrhotic patients admitted to the ICU were initially screened using the MIMIC-IV database. After applying the predefined inclusion criteria, 2998 patients were included in the final cohort for analysis. The baseline characteristics of the cohort revealed a median age of 61 years (IQR 53–68), with males constituting 64.81% of the population. The calculated LHR index had a median value of 8.15 across all participants (IQR: 5.73–13.02). Regarding mortality outcomes, ICU death rates at 30 days, 180 days and 365 days were 29.69%, 34.32% and 35.29%, respectively, as detailed in Table 1.

**Table 1:** Baseline characteristics of the cirrhosis population according to lactate-to-hematocrit ratio quartiles.

Variable	Overall(N = 2998)	Q1(N = 749)	Q2(N = 749)	Q3(N = 749)	Q4(N = 751)	*P*
Demographics						
Age, years	61.00 (53.00–68.00)	62.00 (54.00–71.00)	61.00 (53.00–68.00)	60.00 (52.00–68.00)	60.00 (51.00–67.00)	<.001
Gender: male, n (p%)	1943.00 (64.81%)	499.00 (66.62%)	478.00 (63.82%)	470.00 (62.75%)	496.00 (66.05%)	.348
Weight, kg	82.63 (69.60–99.40)	80.70 (67.50–99.60)	81.80 (69.20–98.30)	82.40 (69.50–98.40)	85.00 (72.60–100.00)	.025
Race, n (p%)						<.001
white	2102.00 (70.11%)	538.00 (71.83%)	551.00 (73.56%)	551.00 (73.56%)	462.00 (61.52%)	
black	241.00 (8.04%)	57.00 (7.61%)	53.00 (7.08%)	53.00 (7.08%)	78.00 (10.39%)	
others	655.00 (21.85%)	154.00 (20.56%)	145.00 (19.36%)	145.00 (19.36%)	211.00 (28.10%)	
Vital signs						
Heart rate, beats/minute	88.13 (77.52–100.19)	83.57 (71.96–94.36)	86.00 (76.04–97.52)	89.09 (78.36–100.41)	95.32 (83.24–106.05)	<.001
MBP, mm Hg	73.21 (66.80–81.05)	74.69 (68.25–83.17)	74.23 (68.22–82.10)	73.67 (66.79–80.90)	70.50 (64.14–77.32)	<.001
RR, breath/minute	18.42 (16.03–21.55)	17.83 (15.77–20.63)	17.90 (15.79–20.81)	18.22 (15.85–20.78)	19.92 (16.91–24.15)	<.001
SpO2, %	97.04 (95.46–98.52)	97.00 (95.49–98.37)	96.96 (95.44–98.48)	97.20 (95.77–98.65)	97.04 (95.12–98.60)	.102
Temperaturef, °F	98.20 (97.80–98.66)	98.23 (97.90–98.70)	98.20 (97.84–98.66)	98.27 (97.87–98.66)	98.10 (97.57–98.60)	<.001
**Score**						
SOFA	9.00 (6.00–12.00)	6.00 (4.00–9.00)	8.00 (5.00–10.00)	9.00 (7.00–11.00)	12.00 (9.00–15.00)	<.001
SAPSII	41.00 (32.00–52.00)	36.00 (28.00–45.00)	39.00 (30.00–48.00)	41.00 (33.00–52.00)	52.00 (41.00–63.00)	<.001
OASIS	33.00 (27.00–40.00)	31.00 (26.00–36.00)	32.00 (26.00–37.00)	33.00 (27.00–39.00)	39.00 (31.00–46.00)	<.001
GCS	15.00 (14.00–15.00)	15.00 (14.00–15.00)	15.00 (14.00–15.00)	15.00 (13.00–15.00)	15.00 (13.00–15.00)	.141
CCI	6.00 (4.00–8.00)	6.00 (4.00–8.00)	6.00 (4.00–8.00)	6.00 (4.00–8.00)	6.00 (4.00–7.00)	0.099
Laboratory data						
LAC, mmol/L	2.30 (1.65–3.53)	1.30 (1.10–1.60)	1.90 (1.70–2.20)	2.74 (2.40–3.15)	5.27 (4.00–8.10)	<.001
HCT, %	27.75 (24.52–31.79)	30.35 (26.90–34.84)	28.10 (25.00–31.95)	26.60 (24.00–30.42)	25.79 (23.23–29.32)	<.001
Hemoglobin, g/dL	9.18 (8.04–10.57)	10.10 (8.70–11.40)	9.38 (8.22–10.70)	8.95 (7.83–10.20)	8.53 (7.60–9.73)	<.001
Platelet, K/uL	100.29 (65.50–151.00)	126.00 (80.00–190.00)	101.18 (69.50–149.20)	92.33 (62.38–136.80)	85.55 (58.60–127.80)	<.001
WBC, K/uL	10.13 (6.70–14.90)	9.15 (6.05–13.30)	9.60 (6.67–14.37)	10.50 (6.93–14.80)	11.50 (7.22–17.50)	<.001
RBC, m/uL	2.97 (2.54–3.43)	3.25 (2.86–3.74)	3.05 (2.60–3.50)	2.83 (2.47–3.28)	2.69 (2.37–3.14)	<.001
Glucose, mg/dL	130.00 (107.00-173.00)	121.00 (102.50–149.00)	127.67 (106.00–160.50)	138.33 (113.20–186.00)	140.67 (108.20–196.80)	<.001
Sodium, mEq/L	137.00 (133.00–140.33)	137.50 (134.50–140.67)	136.75 (133.00–139.57)	137.00 (133.00–140.00)	136.75 (132.33–141.00)	.003
INR	1.70 (1.40–2.20)	1.47 (1.25–1.77)	1.60 (1.40–2.03)	1.80 (1.50–2.20)	2.08 (1.70–2.73)	<.001
PT, sec	18.61 (15.55–23.50)	16.03 (13.70–19.17)	17.60 (15.10–21.85)	19.45 (16.50–23.35)	22.47 (18.70–29.65)	<.001
ALT, IU/L	37.00 (21.00–99.50)	29.33 (17.00–61.00)	35.00 (20.50–73.00)	35.00 (20.67–113.67)	54.00 (26.00–214.00)	<.001
AST, IU/L	80.00 (42.00–215.50)	56.00 (31.00–105.00)	73.00 (40.00–156.00)	82.67 (45.50–238.00)	141.00 (60.00–506.67)	<.001
TBIL, mg/dL	2.92 (1.30–7.23)	1.47 (0.75–3.37)	2.55 (1.30–6.50)	3.50 (1.70–8.10)	5.00 (2.59–11.65)	<.001
Creatinine, mg/dL	1.35 (0.85–2.33)	1.10 (0.75–2.05)	1.20 (0.80–2.00)	1.33 (0.87–2.20)	1.78 (1.18–2.77)	<.001
BUN, mg/dL	28.00 (16.75–47.00)	24.00 (14.00–45.00)	27.00 (17.00–44.00)	29.00 (18.00–46.67)	32.33 (18.50–49.50)	<.001
LHR,%	8.15 (5.73–13.02)	4.52 (3.68–5.15)	6.80 (6.25–7.41)	10.13 (9.06–11.35)	19.48 (15.33–29.49)	<.001
Comorbidities						
Renal failure, n (p%)	3.00 (0.10%)	1.00 (0.13%)	1.00 (0.13%)	1.00 (0.13%)	0.00 (0.00%)	.800
Respiratory failure, n (p%)	14.00 (0.47%)	9.00 (1.20%)	3.00 (0.40%)	2.00 (0.27%)	0.00 (0.00%)	.005
HCC, n (p%)	165.00 (5.50%)	20.00 (2.67%)	33.00 (4.41%)	57.00 (7.61%)	55.00 (7.32%)	<.001
Hepatopulmonary syndrome, n (p%)	44.00 (1.47%)	7.00 (0.93%)	20.00 (2.67%)	11.00 (1.47%)	6.00 (0.80%)	.010
SBP, n (p%)	295.00 (9.84%)	38.00 (5.07%)	63.00 (8.41%)	92.00 (12.28%)	102.00 (13.58%)	<.001
Hepatorenal syndrome, n (p%)	440.00 (14.68%)	49.00 (6.54%)	95.00 (12.68%)	134.00 (17.89%)	162.00 (21.57%)	<.001
GIB, n (p%)	83.00 (2.77%)	27.00 (3.60%)	16.00 (2.14%)	22.00 (2.94%)	18.00 (2.40%)	.318
HF, n (p%)	606.00 (20.21%)	226.00 (30.17%)	161.00 (21.50%)	127.00 (16.96%)	92.00 (12.25%)	<.001
Treatments						
Antibiotics, n (p%)	2792.00 (93.13%)	666.00 (88.92%)	680.00 (90.79%)	715.00 (95.46%)	731.00 (97.34%)	<.001
GC, n (p%)	1097.00 (36.59%)	188.00 (25.10%)	267.00 (35.65%)	308.00 (41.12%)	334.00 (44.47%)	<.001
Clinical outcomes						
30-day mortality, n (p%)	890.00 (29.69%)	123.00 (16.42%)	158.00 (21.09%)	192.00 (25.63%)	417.00 (55.53%)	<.001
180-day mortality, n (p%)	1029.00 (34.32%)	151.00 (20.16%)	187.00 (24.97%)	237.00 (31.64%)	454.00 (60.45%)	<.001
365-day mortality, n (p%)	1058.00 (35.29%)	160.00 (21.36%)	192.00 (25.63%)	242.00 (32.31%)	464.00 (61.78%)	<.001

ALT = alanine aminotransferase, AST = aspartate aminotransferase, BUN = blood urea nitrogen, CCI = charlson comorbidity index, GC = glucocorticoids, GCS = glasgow coma scale, GIB = gastrointestinal bleeding, HCC = hepatocellular carcinoma, HCT = hematocrit, HF = heart failure, INR = international normalized ratio, LAC = lactate, LHR = lactate-to-hematocrit ratio, MBP = mean blood pressure, OASIS = Oxford acute severity of illness score, PT = prothrombin time, RBC = red blood cells, RR = respiratory rate, SAPS II = simplified acute physiology score II, SBP = spontaneous bacterial peritonitis, SOFA = sequential organ failure assessment score, SpO_2_ = percutaneous arterial oxygen saturation, TBIL = total bilirubin, WBC = white blood cell.

### 3.1. Study population baseline characteristics

Table 1 summarizes the baseline characteristics of critically ill cirrhotic patients categorized by LHR quartiles. The cohort was divided into 4 groups based on LHR values at hospital admission, with the following ranges: Q1: 1.145 to 5.729; Q2: 5.729 to 8.146; Q3: 8.148 to 12.991; Q4: 13 to 136.765. The median LHR values for each quartile were 4.52 (IQR: 3.68–5.15), 6.80 (IQR: 6.25–7.41), 10.13 (IQR: 9.06–11.35) and 19.48 (IQR: 15.33–29.49), respectively. Patients in the highest LHR quartile (Q4) exhibited several distinguishing features: they were generally younger, had higher body weight and included a lower proportion of Caucasians. Vital sign assessments revealed that this group had elevated heart and respiratory rates, along with reduced blood pressure and body temperature. Disease severity scores, including SOFA, SAPS II and OASIS, progressively increased with higher LHR values. Laboratory findings indicated that the Q4 group showed elevated levels of WBC, glucose, INR, PT, ALT, AST, TBIL and BUN, whereas hemoglobin, platelet count, RBC and sodium levels were lower. Regarding comorbidities, the Q4 group had higher incidence rates of HCC, SBP and hepatorenal syndrome, but lower frequencies of respiratory failure, hepatopulmonary syndrome and HF. In terms of clinical interventions, patients in the highest LHR quartile received antibiotics and GC more frequently. Consequently, clinical outcome analysis demonstrated that these patients also experienced the highest all-cause mortality at 30 days, 180 days and 365 days of follow-up.

### 3.2. Association between LHR and mortality in critically ill cirrhosis patients

**Figure 2.**
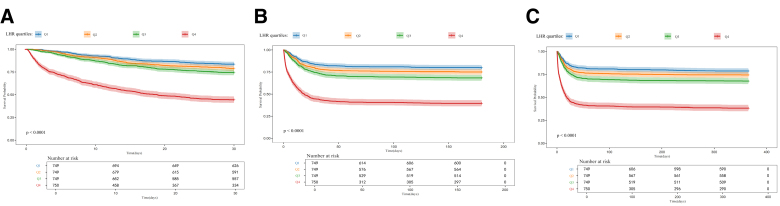
Kaplan–Meier according to LHR quartiles (A, 30-day mortality; B, 180-day mortality; C, 365-day mortality; LHR, lactate-to-hematocrit ratio).

Significant disparities in short- and long-term survival stratified by LHR quartiles were observed in Kaplan–Meier curves (Fig. 2). Notably, the highest LHR group exhibited the poorest survival probability throughout the 30-day, 180-day and 365-day follow-ups (log-rank test, *P* < .001).

To evaluate the association of the LHR index with all-cause mortality in critically ill patients with cirrhosis, we performed multivariate Cox regression analyses, as summarized in Table 2. The multivariable Cox model was informed by both univariate screening (Supplementary Table S1) and clinical expertise. The results indicated that elevated LHR values were significantly associated with higher mortality at 30, 180 and 365 days. In the unadjusted Cox regression (Model 1), higher LHR was strongly linked to an increased risk of death at 30 days (hazard ratio [HR] = 4.87, 95% confidence interval [CI]: 3.982–5.957, *P* < .001), 180 days (HR = 4.499, 95% CI: 3.741–5.41, *P* < .001) and 365 days (HR = 4.375, 95% CI: 3.654–5.239, *P* < .001). After adjustment for age, gender, weight and race in Model 2, the association remained statistically significant, with persistently elevated risks observed for 30-day (HR = 5.055, 95% CI: 4.127–6.192, *P* < .001), 180-day (HR = 4.649, 95% CI: 3.86–5.598, *P* < .001) and 365-day mortality (HR = 4.516, 95% CI: 3.767–5.415, *P* < .001). Further adjustments were made in Model 3 for a comprehensive set of clinical confounders, including age, gender, race, MBP, SOFA score, OASIS score, creatinine, glucose, INR, sodium, WBC, TBIL, hepatorenal syndrome, SBP and antibiotic use. Even under this extensively adjusted model, higher LHR remained an independent predictor of all-cause mortality. Moreover, a significant trend of increasing mortality risk was observed across ascending LHR quartiles (P for trend < 0.001).

**Table 2:** The association between lactate-to-hematocrit ratio and all-cause mortality.

	Model 1		Model 2		Model 3	
	HR (95%CI)	*P*	HR (95%CI)	*P*	HR (95%CI)	*P*
30-day mortality						
LHR	1.052(1.048,1.055)	<.001	1.052 (1.048,1.055)	<.001	1.034 (1.029,1.038)	<.001
LHR quartiles						
Q1	ref		Ref		ref	
Q2	1.317 (1.041,1.667)	.022	1.357 (1.071,1.718)	.011	1.116 (0.88,1.417)	.366
Q3	1.652 (1.318,2.072)	<.001	1.71 (1.363,2.145)	<.001	1.237 (0.98,1.561)	.073
Q4	4.87 (3.982,5.957)	<.001	5.055 (4.127,6.192)	<.001	2.442 (1.951,3.057)	<.001
P for trend	<.001		<.001		<.001	
180-day mortality						
LHR	1.051 (1.048,1.055)	<.001	1.051 (1.048,1.055)	<.001	1.034 (1.03,1.039)	<.001
LHR quartiles						
Q1	ref		Ref		ref	
Q2	1.277(1.031,1.582)	.025	1.31 (1.057,1.623)	.014	1.089 (0.877,1.352)	.442
Q3	1.682 (1.372,2.063)	<.001	1.733 (1.413,2.127)	<.001	1.277 (1.035,1.576)	.023
Q4	4.499 (3.741,5.41)	<.001	4.649 (3.86,5.598)	<.001	2.396 (1.95,2.944)	<.001
*P* for trend	<.001		<.001		<.001	
365-day mortality						
LHR	1.051 (1.048,1.055)	<.001	1.051 (1.048,1.055)	<.001	1.035 (1.03,1.039)	<.001
LHR quartiles						
Q1	ref		Ref		ref	
Q2	1.238 (1.004,1.528)	.046	1.267 (1.027,1.564)	.027	1.059 (0.856,1.309)	.597
Q3	1.625 (1.331,1.984)	<.001	1.672 (1.369,2.043)	<.001	1.247 (1.014,1.533)	.036
Q4	4.375 (3.654,5.239)	<.001	4.516 (3.767,5.415)	<.001	2.391 (1.955,2.925)	<.001
*P* for trend	<.001		<.001		<.001	

Model 1: no adjustment. Model 2: adjust for age, gender, weight, and race. Model 3: adjust for age, gender, race, MBP, SOFA, OASIS, creatinine, glucose, INR, sodium, WBC, TBIL, hepatorenal syndrome, SBP, and antibiotics. CI = confidence interval, HR = hazard ratio, INR = international normalized ratio, LHR = lactate-to-hematocrit ratio, MBP = mean blood pressure, OASIS = oxford acute severity of illness score, SBP = spontaneous bacterial peritonitis, SOFA = sequential organ failure assessment score, TBIL = total bilirubin, WBC = white blood cell.

### 3.3. Nonlinear relationships between LHR and mortality

**Figure 3.**
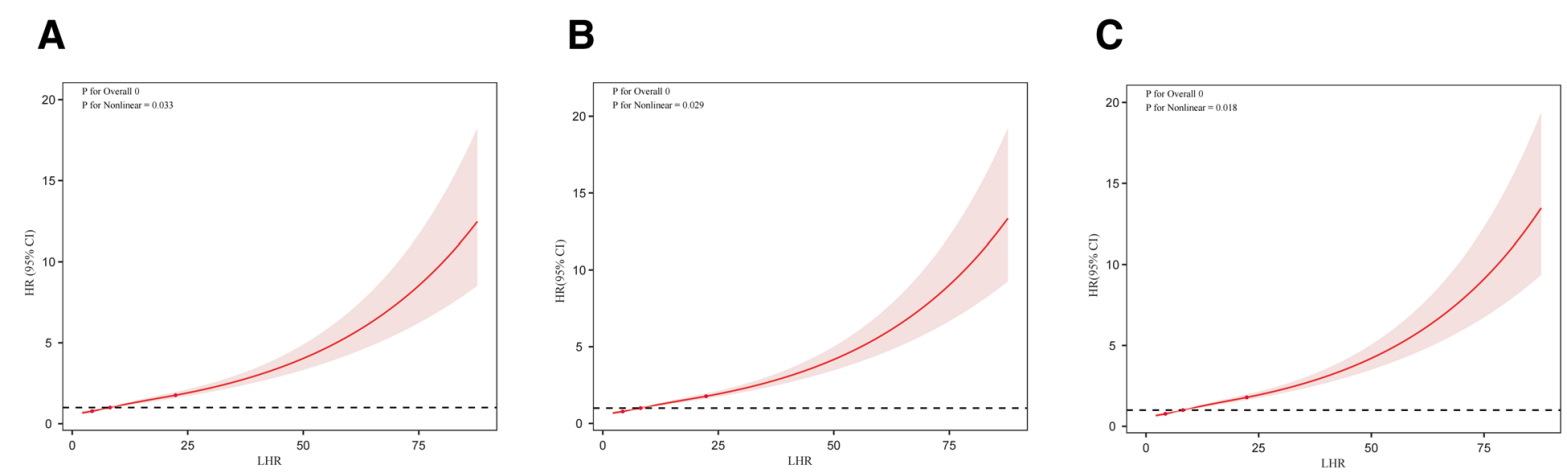
RCS analysis of mortality risk with LHR in cirrhosis (A, 30-day mortality; B, 180-day mortality; C, 365-day mortality; LHR, lactate-to-hematocrit ratio).

RCS analysis revealed a nonlinear association between LHR and all-cause mortality at 30 (*P* for nonlinear = .033), 180 (*P* for nonlinear = .029) and 365 days (*P* for nonlinear = .018) in critically ill patients with cirrhosis, as visualized in Figure 3.

### 3.4. Subgroup analysis

**Figure 4.**
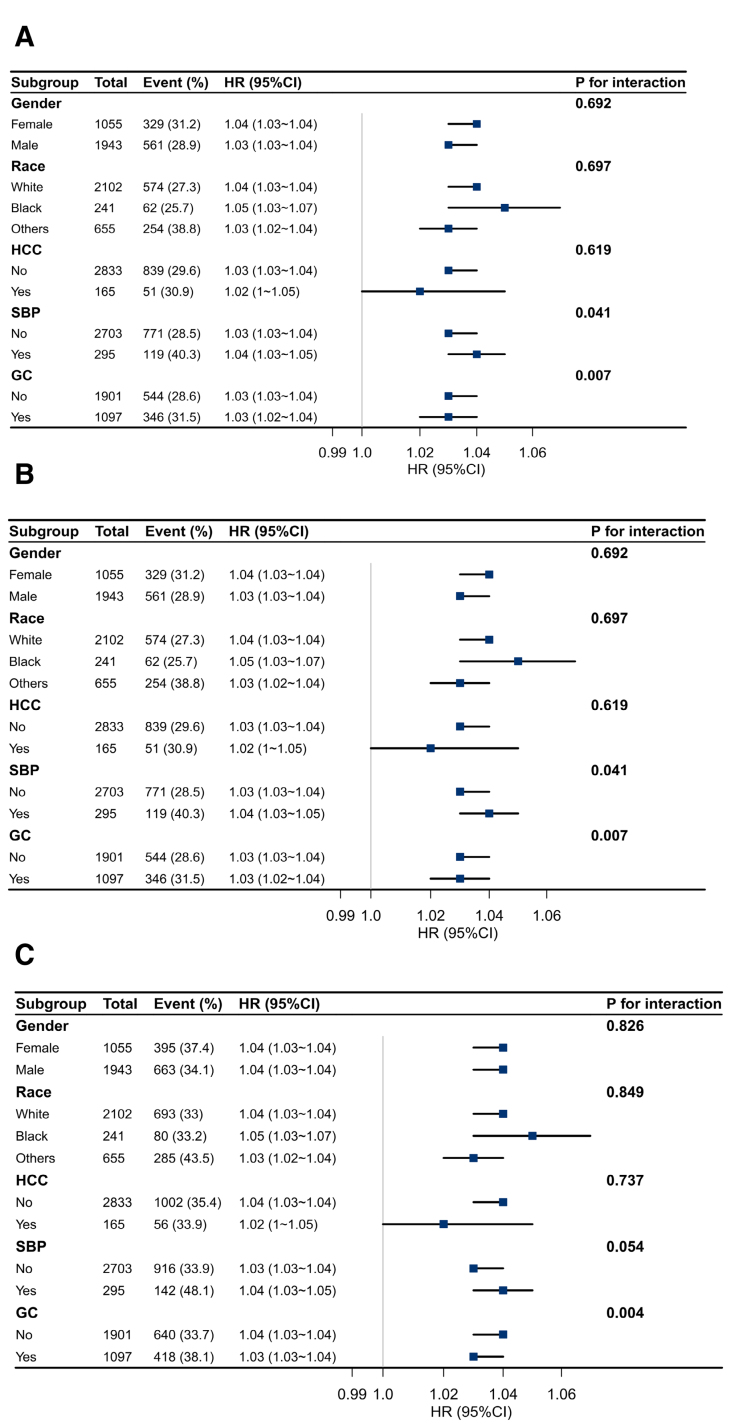
Subgroup analysis of the association between LHR and mortality risk (A, 30-day mortality; B, 180-day mortality; C, 365-day mortality. HCC, hepatocellular carcinoma; SBP, spontaneous bacterial peritonitis; GC, glucocorticoids).

Subgroup analyses indicated that the positive association between elevated LHR and higher mortality was consistent in most predefined subgroups, such as those based on gender, race and HCC history (all interaction *P* > .05). However, the effect of LHR was significantly modified by the presence of SBP and the use of GC. This pattern of interaction was evident in both the 30-day and 180-day mortality analyses, while the interaction with GC use remained statistically significant in the 365-day analysis (Fig. 4).

## 4. Discussion

Advanced liver cirrhosis, a terminal-stage condition frequently seen in the ICU, poses a considerable global health challenge due to its high mortality.^[31,32]^ It accounted for 2.4% of worldwide deaths in 2019, ranking as the seventh leading cause among middle-aged and elderly groups.^[33,34]^ The clinical course of these critically ill patients is often marked by rapid deterioration, underscoring the need for reliable prognostic tools. While various markers, from invasive measures like liver biopsy to scoring systems such as MELD, have been proposed, their application is hampered by limitations including complexity, subjectivity and poor reproducibility.^[35–37]^ Therefore, the timely prognosis and risk stratification of critically ill cirrhotic patients continue to confront significant obstacles.

This study investigated the prognostic value of LHR in critically ill cirrhotic patients. Analysis of data from the MIMIC-IV database showed that elevated LHR levels were strongly linked to an increased risk of mortality. Consistent findings were observed across Kaplan–Meier survival curves and multivariate Cox regression models, which demonstrated that higher LHR quartiles significantly increased mortality risk at 30, 180 and 365 days. Additionally, RCS analysis revealed a significant nonlinear relationship between LHR levels and mortality risk.

Synthesized under conditions of tissue hypoxia, lactate (LAC) is primarily cleared by the liver under physiological conditions, thus playing a key role in sustaining metabolic stability.^[14]^ In cirrhotic patients, impaired hepatic clearance capacity results in lactate accumulation, thereby inducing acid-base imbalance and metabolic disturbances. Notably, elevated levels of circulating lactate have been listed as an important predictor of increased mortality in this patient population.^[20,38,39]^ HCT, which denotes the volume percentage of erythrocytes in blood, is modulated by inflammation, oxidative stress, hemorrhage, fluid balance shifts and iatrogenic hemodilution. Depressed HCT levels, commonly observed in cirrhotic patients due to liver dysfunction, malnutrition, hypersplenism and anemia,^[10,25]^ are recognized predictors of poor prognosis.^[25,28]^ Independently, however, the interpretation of both HCT and LAC remains complex. While LAC elevation in cirrhosis can result from deficient hepatic clearance, routinely measured arterial LAC does not discriminate its origin, whether metabolic impairment or systemic hypoperfusion. Likewise, although HCT informs on blood viscosity and oxygen transport, diminished levels in cirrhosis are closely tied to anemia, inadequate oxygen supply and related metabolic dysregulation.^[40]^ The LHR has recently emerged as a promising composite biomarker that concurrently reflects disturbances in oxygen metabolism and states of hemodilution.^[9,10]^ Its value in predicting outcomes has been increasingly validated in diverse critical care populations. For instance, one investigation^[10]^ established LHR as an independent determinant of 30-day mortality in critically ill patients, showing better predictive accuracy than either LAC or HCT alone. Among critically ill individuals with cirrhosis, elevated LAC can result not only from systemic hypoperfusion but also from reduced hepatic clearance and underlying metabolic dysregulation.^[17–19,41]^ Meanwhile, HCT serves as an indicator of hemoconcentration or dilutional status, variations in circulating blood volume and the presence and severity of anemia. A decline in HCT can further impair tissue oxygen delivery and its co-occurrence with high LAC levels often signals a progressive mismatch between systemic oxygen supply and demand.^[25]^ By integrating both LAC and HCT into a unified index, LHR provides a composite measure that captures the extent of oxygenation failure and overall illness severity. This integrated approach provides a more comprehensive evaluation of circulatory status, tissue perfusion and metabolic condition, thereby improving the accuracy of pathophysiological assessment in complex clinical settings. To our knowledge, this work represents the first investigation to establish the prognostic significance of LHR specifically in critically ill cirrhotic patients. The findings endorse LHR as a practical prognostic tool in this population, where elevated values robustly correlate with poorer outcomes across both short- and long-term follow-up. These observations align with earlier studies that reported the predictive utility of LHR in other critical illness contexts.^[9–11]^

The prognostic association of LHR was found to vary significantly across patient subgroups, with notable effect modifications related to SBP and GC use. Specifically, SBP showed significant interactions at 30 and 180 days, while GC therapy exhibited a persistent interaction throughout the entire follow-up period. SBP, a common and severe complication of cirrhosis, induces a robust systemic inflammatory response that exacerbates the patient’s metabolic stress. In the context of SBP, lactate accumulation is not merely a result of hepatic dysfunction but also reflects the additional metabolic and inflammatory burden induced by infection. Elevated lactate levels in SBP are linked to impaired hepatic lactate clearance and hypoperfusion due to sepsis-induced circulatory failure, which further compromises oxygen delivery and tissue perfusion. This complex interplay amplifies the prognostic value of LHR, particularly in the short term, where elevated LHR indicates a heightened risk of mortality due to the compounded effects of infection and metabolic dysregulation. Therefore, clinicians should be aware that elevated LHR in the presence of SBP may signal an exacerbation of critical illness, requiring urgent intervention to address both infection and circulatory failure. On the other hand, the use of GC, often administered to manage inflammation and acute liver failure in cirrhotic patients, introduces additional complexity in interpreting LHR. While corticosteroids reduce systemic inflammation, they also induce metabolic changes, including altered glucose metabolism and fluid shifts, which affect both lactate production and hematocrit levels. This makes the interpretation of elevated LHR in GC-treated patients more challenging, as the lactate levels may not solely reflect impaired tissue oxygenation or hepatic dysfunction, but also the metabolic derangements induced by steroid therapy. The effect of GC on the immune response and metabolism could modify the predictive value of LHR, especially in the long term, making it a less reliable marker in patients under prolonged steroid treatment. Thus, interpreting elevated LHR in GC-treated patients requires careful consideration of the immunomodulatory effects of corticosteroids. LHR serves as a practical tool to refine prognostic evaluation and personalize treatment in cirrhotic patients. Elevated levels should prompt clinicians to consider earlier escalation of care, such as optimizing oxygen delivery, correcting hemodynamic instability and addressing underlying causes of hyperlactatemia. These steps may help reduce metabolic stress and improve recovery potential. Additionally, since high LHR often coincides with hemodynamic compromise, individualized fluid management is essential. Interpreting LHR in the context of the patient’s volume status may inform whether fluid restriction or augmentation is indicated, thus minimizing the risks of both overload and depletion and supporting better therapeutic precision.

This investigation possesses several distinct strengths. It represents the first comprehensive assessment linking LHR to both short- and long-term prognosis incirrhotic patients. In contrast to conventional multiparameter scoring systems, LHR is derived from routinely collected laboratory and monitoring variables, eliminating the need for complex calculations. This accessibility enhances its utility for rapid risk stratification, particularly in resource-constrained or time-critical environments. Moreover, the use of high-quality, real-world data from the MIMIC-IV (v3.1) database allowed for extensive statistical adjustment, strengthening the validity of the findings by minimizing potential confounding. In summary, LHR functions as a novel prognostic biomarker that combines predictive accuracy with operational simplicity. Its objective and readily calculable nature facilitates timely clinical assessment, supports the formulation of individualized treatment strategies and may ultimately contribute to improved patient survival and outcomes.

This study has several important limitations that should be acknowledged. First, although the analysis was based on the high-quality MIMIC-IV database, the cohort represents critically ill patients admitted to a single tertiary-care ICU in the United States. Consequently, the findings may not be fully generalizable to cirrhotic patients managed outside the ICU or in healthcare systems with different resource availability. Future multicenter studies across diverse clinical settings are needed to confirm the external validity of our results. Second, the retrospective observational design precludes causal inference. While robust associations between LHR and mortality were observed after multivariable adjustment, residual confounding cannot be completely excluded. Prospective studies are warranted to clarify the causal pathways linking metabolic stress, hematologic alterations, and adverse outcomes in critically ill cirrhotic patients. Third, only the first ICU admission was included for each patient. This approach may have introduced selection bias by excluding patients with recurrent or prolonged ICU courses, who may represent a distinct and potentially higher-risk subgroup. Longitudinal studies incorporating repeated ICU admissions could provide additional insight into disease trajectory and risk accumulation. Fourth, despite adjustment for multiple clinical and laboratory variables, unmeasured confounders such as alcohol consumption patterns, nutritional status, genetic susceptibility, and social determinants of health were not captured in the database and may have influenced both LHR levels and clinical outcomes. Incorporation of these factors in future studies may further refine risk stratification. Fifth, LHR was calculated using laboratory values obtained within the first 24 hours of ICU admission, which does not account for dynamic changes during the course of critical illness. Given the fluctuating nature of metabolic and hemodynamic states in cirrhosis, future research should evaluate longitudinal LHR trajectories to determine whether temporal patterns offer incremental prognostic value beyond baseline measurements. Finally, the diagnosis of cirrhosis relied on ICD-9/10 codes, which may introduce misclassification bias and may not fully capture disease etiology or severity. Validation using clinically adjudicated diagnoses or imaging- and biopsy-based criteria would strengthen future investigations.

## 5. Conclusion

LHR is a reliable and readily available biomarker that independently predicts both short- and long-term all-cause mortality in critically ill patients with cirrhosis. Its integration into early clinical assessment may facilitate timely risk stratification, support individualized clinical decision-making, and improve the allocation of intensive care resources. Future prospective, multicenter studies are warranted to validate these findings and to further explore the utility of LHR for dynamic monitoring during critical illness.

## Acknowledgments

The authors gratefully acknowledge the MIMIC team for developing and maintaining the MIMIC-IV database and for providing open access to this invaluable resource. We also sincerely thank Professor Yuan Yuan for her expert guidance and substantial assistance in medical scientific writing and language refinement, which greatly improved the clarity and readability of this manuscript.

## Author contributions

**Conceptualization:** Ya Chen, Yawen Luo.

**Data curation:** Yunfen Chen, Yawen Luo.

**Formal analysis:** Longmin Qiu

**Investigation:** Yihuai He, Yunfen Chen, Longmin Qiu.

**Methodology:** Ya Chen, Yihuai He.

**Software:** Yunfen Chen.

**Writing – original draft:** Ya Chen.

**Writing – review & editing:** Yawen Luo


