# Supplementary material for: Risk analysis of the association between lactate-to-hematocrit ratio and poor prognosis in critical patients with cirrhosis
Source: Medicine (Baltimore). 2026 Jun 5;105(23):e49155. doi: 10.1097/MD.0000000000049155 (PMC13246079; doi:10.1097/MD.0000000000049155)
Supplement: Supplementary file 1 [file medi-105-e49155-s001.docx]

Table S1 **Univariate Cox regression analysis of ICU all-cause mortality rate at 30 days**

| **Variables** | **HR (95%CI)** | ***P*** |
| --- | --- | --- |
| **Demographics** | | |
| Age | 1.006 (1.001–1.011) | 0.025 |
| Gender |  |  |
| Female | 1.00 (Reference) |  |
| Male | 0.923 (0.806–1.058) | 0.252 |
| Weight | 1.003 (1.001–1.006) | 0.019 |
| Race |  |  |
| white | 1.00 (Reference) |  |
| black | 0.937 (0.721–1.218) | 0.629 |
| others | 1.551 (1.338–1.798) | <0.001 |
| **Vital signs** | | |
| Heart rate | 1.019 (1.015–1.023) | <0.001 |
| MBP | 0.967 (0.961–0.973) | <0.001 |
| RR | 1.097 (1.082–1.111) | <0.001 |
| Temperaturef | 0.984 (0.974–0.995) | 0.003 |
| **Score** | | |
| SOFA | 1.208 (1.19–1.227) | <0.001 |
| SAPSII | 1.05 (1.046–1.054) | <0.001 |
| OASIS | 1.075 (1.067–1.082) | <0.001 |
| **Laboratory data** | | |
| LAC | 1.217 (1.201–1.233) | <0.001 |
| HCT | 0.98 (0.968–0.993) | 0.002 |
| Hemoglobin | 0.923 (0.889–0.959) | <0.001 |
| Platelet | 0.999 (0.998–1) | 0.058 |
| WBC | 1.019 (1.015–1.022) | <0.001 |
| RBC | 0.694 (0.623–0.774) | <0.001 |
| Glucose | 0.997 (0.996–0.999) | 0.999 |
| Sodium | 0.987 (0.976–0.998) | 0.018 |
| INR | 1.552 (1.481–1.627) | <0.001 |
| PT | 1.04 (1.036–1.045) | <0.001 |
| ALT | 1 (1–1) | 0.559 |
| AST | 1 (1–1) | <0.001 |
| TBIL | 1.037 (1.032–1.043) | <0.001 |
| Creatinine | 1.175 (1.143–1.208) | <0.001 |
| BUN | 1.011 (1.009–1.013) | <0.001 |
| LHR | 1.052 (1.048–1.055) | <0.001 |
| **Comorbidities** | | |
| Respiratory failure |  |  |
| No | 1.00 (Reference) |  |
| Yes | 1.231 (0.511–2.964) | 0.644 |
| HCC |  |  |
| No | 1.00 (Reference) |  |
| Yes | 1.068 (0.805–1.418) | 0.646 |
| Hepatopulmonary syndrome |  |  |
| No | 1.00 (Reference) |  |
| Yes | 0.846 (0.479–1.495) | 0.565 |
| SBP |  |  |
| No | 1.00 (Reference) |  |
| Yes | 1.472 (1.214–1.785) | <0.001 |
| Hepatorenal syndrome |  |  |
| No | 1.00 (Reference) |  |
| Yes | 1.724 (1.471–2.02) | <0.001 |
| HF |  |  |
| No | 1.00 (Reference) |  |
| Yes | 0.969 (0.822–1.142) | 0.704 |
| **Treatments** | | |
| Antibiotics |  |  |
| No | 1.00 (Reference) |  |
| Yes | 2.34 (1.616–3.389) | <0.001 |
| GC |  |  |
| No | 1.00 (Reference) |  |
| Yes | 1.094 (0.956–1.252) | 0.189 |

Abbreviation: MBP, mean blood pressure; RR, respiratory rate; SOFA, sequential organ failure assessment score; SAPS II, simplified acute physiology score II; OASIS, oxford acute severity of illness score; LAC, lactate; HCT, hematocrit; WBC, white blood cell; RBC, red blood cell; INR, international normalized ratio; PT, prothrombin time; ALT, alanine aminotransferase; AST, aspartate aminotransferase; TBIL, total bilirubin; BUN, blood urea nitrogen; LHR, lactate-to-hematocrit ratio; HCC, hepatocellular carcinoma; SBP, spontaneous bacterial peritonitis; HF, heart failure; GC, glucocorticoids.
